# Not Always (and Only) Heart Failure—A Case Report of Primary Pleural Lymphoma in an Elderly Patient

**DOI:** 10.3390/clinpract11010006

**Published:** 2021-01-29

**Authors:** Tiago Rabadão, Leonor Naia, Filipa Ferreira, Mariana Teixeira, Marcelo Aveiro, Margarida Eulálio, Fernando Silva

**Affiliations:** 1 Medicine Department, Centro Hospitalar do Baixo Vouga, Artur Ravara, 3810-164 Aveiro, Portugal; lnaiagoncalves@gmail.com (L.N.); afilipa.csferreira@gmail.com (F.F.); marianadealmeidateixeira@gmail.com (M.T.); marceloaveiro90@gmail.com (M.A.); meulalio@hotmail.com (M.E.); 2 Hematology Department, Centro Hospitalar do Baixo Vouga, Artur Ravara, 3810-164 Aveiro, Portugal; Fernando9191@yahoo.com.br

**Keywords:** pleural primary lymphoma, hematologic neoplasms, pleural effusion, thoracentesis, population-ageing

## Abstract

Pleural involvement in Non-Hodgkin Lymphoma (NHL) is well documented, but primary pleural lymphomas are extremely rare, occurring mostly in immunosuppressed patients or associated with chronic pleural inflammation. Nevertheless, the pathogenesis and therapeutic approaches to counteract primary pleural lymphomas are still matter of debate. The authors present the clinical case of an 81-year-old female with respiratory and constitutional symptoms. A valvular heart disease and bilateral pleural effusion were known. The study carried out showed a large right pleural effusion; the fluid analysis was compatible with Diffuse Large B-cell Lymphoma (DLBCL), and two lymphomatous masses with pleural origin were found at the ipsilateral hemithorax. Primary pleural lymphoma was considered and chemotherapy was initiated with a good response and evolution. The authors report this remarkable clinical case because of its rarity, its excellent clinical evolution and the absence of an immunodeficiency context.

## 1. Introduction

Pleural effusion is a common clinical complication of heart failure, a burden clinical syndrome in elderly patients; differential diagnosis must also include infections and cancer. Hematologic neoplasms should be considered as cause of malignant pleural effusion, even though less common [1,2]. Diffuse Large B-cell Lymphoma (DLBCL), the most common type of Non-Hodgkin Lymphoma (NHL) worldwide, frequently presents with extranodal locations [3], including in the pleura at an advanced stage. The presence of pleural effusion at the time of presentation of lymphoma, is associated with extremely poor outcomes [4,5]. However, primary pleural lymphomas without other secondary organ involvement are extremely rare (less than 1% of all lymphomas) [6], and therefore the risk of misdiagnosis is high [7]. In the majority of cases, primary pleural lymphomas occur in immunosuppressed patients or associated with chronic pleural inflammation [8]. The pathogenesis and therapeutic options are still controversial, and subject of debate.

## 2. Case Presentation

**Figure 1:**
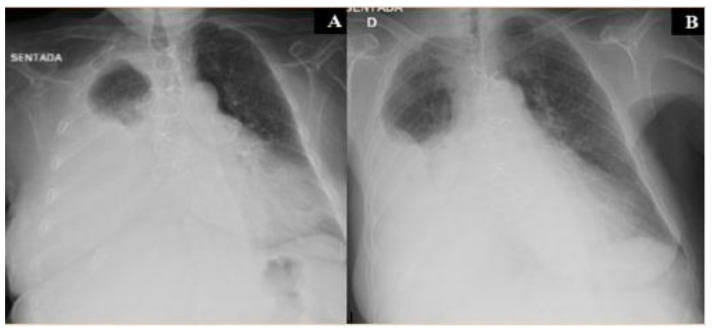
Thoracic X-Ray showing increased cardiac index and a large right pleural effusion, before thoracentesis (**A**); Thoracic X-Ray showing less prominent right pleural effusion, after thoracentesis (**B**).

**Figure 2:**
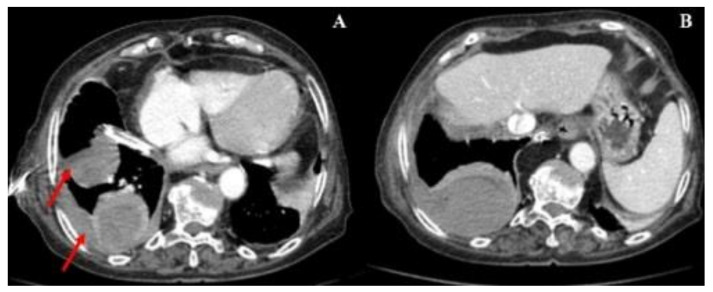
Thoracic-abdominal-pelvic CT showing two lymphomatous masses in right superior lobe with pleural origin (red arrows); no splenomegaly or intra-abdominal and mediastinal lymphadenopathies (**A**); Thoracic-abdominal-pelvic CT showing right pleural effusion, after thoracentesis and intravenous diuretic therapy (**B**).

**Figure 3:**
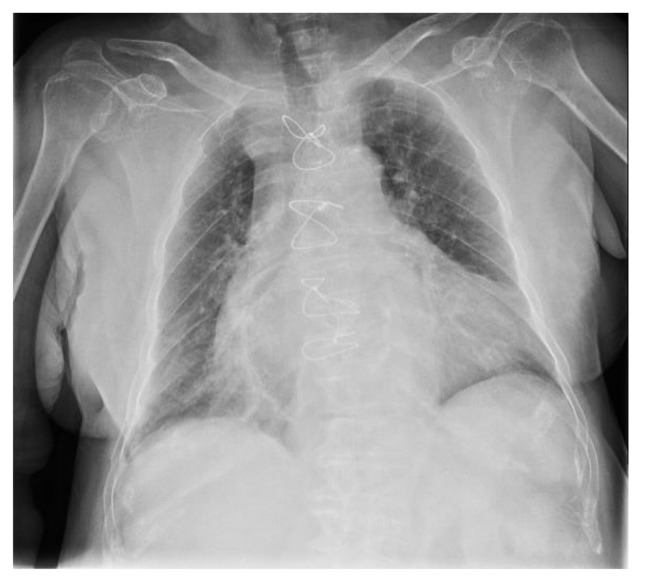
Thoracic X-Ray, performed after eight cycles of chemotherapy, showing radiological improvement, without right pleural effusion.

**Figure 4:**
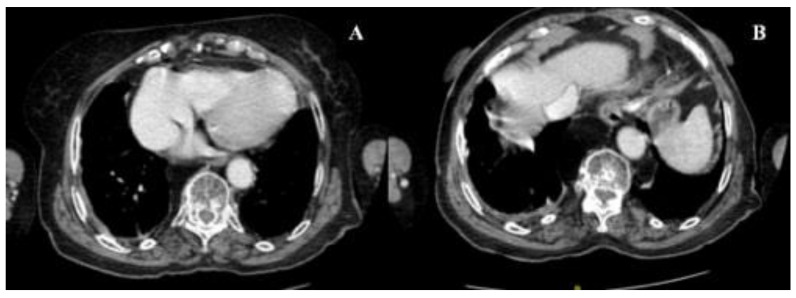
Thoracic-abdominal-pelvic CT, performed after eight cycles of chemotherapy, showing radiological improvement, without lymphomatous masses in right superior lobe (**A**) or right pleural effusion (**B**).

An 81-year-old female presented with a 2-week history of dyspnea, fatigue, anorexia and weight loss (not quantified); respiratory symptoms had not improved with increasing doses of diuretics over the last days. The patient performance status (PS) on the scale of Eastern Cooperative Oncology Group (ECOG) was three. Her significant past medical history included: valvular heart disease (NYHA III class), with severe aortic stenosis replacement valve surgery 2 months before; bilateral pleural effusion, mainly right sided; atrial fibrillation; chronic renal disease; metabolic syndrome and constipation. No neoplasm, smoking or occupational exposure history was reported. Usual medication included: acenocumarol 4 mg, furosemide 40 mg, spironolactone 25 mg, lisinopril 2.5 mg, digoxin 0.125 mg and atorvastatin 10 mg. At admission, the patient was afebrile, eupnoeic with a peripheral oxygen saturation of 84% (FiO2 21%), blood pressure at 86/48 mmHg and heart rate of 78 bpm; thoracic auscultation revealed irregular cardiac sounds with an holosystolic mitral murmur and vesicular sound absent in the lower 2/3 of the right pulmonary hemithorax. No lower limb oedema, lymphadenopathies or abdominal abnormalities were present. The initial analytical workup showed: elevated lactate dehydrogenase —514 U/L (120–246 U/L), elevated brain-type natriuretic peptide —630 pg/mL (<100 pg/mL), elevated β2-Microglobulin —4010 ng/mL (<3000 ng/mL); normal complete blood count, normal renal, hepatic and thyroid function and non-elevated inflammatory parameters. The thoracic X-Ray revealed an increased cardiac index and a large right pleural effusion (Figure 1A); transthoracic echocardiogram showed a severe mitral regurgitation and a left ventricular function of 55%. A thoracentesis was performed (Figure 1B): serohematic pleural fluid was drained, an exudate according to Light criteria (proteins 4.9 g/dL, lactate dehydrogenase 3724 U/L, glucose 30 mg/dL, pH 7.09); cultural and Mycobacterium tuberculosis assay were negative; blood-cell count was high, mainly due to polymorphonuclear leukocytes (9715 cells/mcL) and small and medium mononuclear cells (7733 cells/mcL), with atypia; flow cytometry immunophenotyping (BD FACSCantoIITM) was compatible with DLBCL (54% of clonal B-cells, CD20+). Thoracic-abdominal-pelvic Computed Tomography (CT) showed two lymphomatous masses in the right superior lobe with pleural origin; no splenomegaly or intra-abdominal and mediastinal lymphadenopathies were found (Figure 2). Bone marrow involvement, infectious and autoimmune diseases were ruled out. Primary pleural lymphoma was considered and the patient started chemotherapy with Cyclophosphamide and Prednisolone (due to lack of tolerability with regimen of rituximab, cyclophosphamide, vincristine and prednisolone (R-CVP), first-line treatment); a chest tube was also placed, followed by pleurodesis; oxygen and intravenous diuretic therapy were also needed. After eight cycles of chemotherapy, the patient showed clinical and radiological improvement (Figure 3 and Figure 4). After three years, the patient maintains a close follow-up in Hematology consultation and no evidence of disease recurrence.

## 3. Discussion

The authors report this remarkable clinical case because of its rarity, its excellent clinical evolution (without first-line treatment) and also the absence of immunodeficiency. The most common type of NHL involving pleural cavity is DLBCL, including in localized forms; low-grade lymphoma, including Mucosa-associated lymphoid tissue (MALT) lymphoma, is also a possible cause [9]. Disease presentations include pleural nodules, pleural thickening or effusion, isolated or in combination [10]. Two subtypes of primary pleural lymphoma were described in literature—Primary effusion lymphoma (associated with human herpesvirus-8 or Epstein barr infection) and Pyothorax- associated lymphoma (associated with Epstein barr) [11]; this case shared none of these features. We highlight the patient advanced age and past medical history, specifically possible role of chronic inflammatory effects of long-standing pleural disease (due to heart failure) in the pathogenesis of neoplasms and also the deleterious age effects in both immunity and therapeutic options. Unspecified clinical presentation, heart disease burden and population-ageing could also lead to misdiagnosis in these cases. Thoracentesis was mandatory to rule out surgical related complications (hemothorax, infection or post-thoracotomy idiopathic effusion) and to perform complementary evaluation. In this report, pleural fluid analyses showed multifactorial mechanisms involved: polymorphonuclear cells were present probably due to cardiogenic source, mainly the surgical procedure performed weeks before, and mononuclear cells, the low pH and glucose level related to the malignant disease [1]. Above all, immunophenotype analysis was crucial to establish a diagnosis. Although tumor anatomopathological of a pleural mass lesion examination is essential to make a firm diagnosis, it was not performed, motivated by the risks associated to this procedure and the high reliability of noninvasive immunophenotype analyses [2]. The subtype of NHL cannot be safely determined, but pleural MALT lymphoma is a more common diagnostic consideration coupled to complete and durable clinical remission with cyclophosphamide and steroids only. Positron emission tomography would be more effective in excluding other localizations, but it was not executed even after treatment because of apparent remission of the tumor as evaluated by CT, and good clinical evolution.

## 4. Conclusions

The authors report an uncommon form of lymphoproliferative disease in which management differed from available guidelines, not only in terms of the diagnostic method but also of the therapeutic approach undertook. Considering age, PS, evidence from pleural effusion examination and good clinical evolution, was reasonable to avoid more invasive diagnostic techniques. The approach to this dilemma could be imposed more frequently in the future due to population ageing. This clinical report highlights that clinical judgment must be privileged, avoiding unnecessary diagnostic procedures, mainly in the elderly. The rarity of this clinical condition requires the exclusion of common infectious etiologies and neoplasms in other localizations.
